# Fly Movie Theater Reveals Secrets of How Insects See the World

**DOI:** 10.1371/journal.pbio.0030209

**Published:** 2005-05-17

Seeing the world pass by as you're moving is a complex feat. In flies, images moving across the eye—so-called optic flow—provide sensory information to neurons to guide behaviors like avoiding obstacles and chasing down mates. This maneuverability, combined with a fast image processing speed and the ease of examining fly neurons, has made flies a popular model for dissecting how this occurs. But since much of what we know about how neurons process visual information comes from static situations—for example, placing a fly in a fixed position and presenting it with moving images—the question of how these processes naturally occur is largely unexplored.

In a new study, a German and a Dutch group headed by Martin Egelhaaf and Roland Kern and by Hans van Hateren, respectively, joined forces to take the analysis of optic flow in the blowfly one step closer to the natural situation. To do this, they used a “panoramic virtual reality stimulator” to show a fly in the laboratory what it would see while flying in nature. The authors combined this with measurements of the response of a motion-sensitive blowfly neuron, called the horizontal system equatorial (HSE) cell.

The traditional model holds that the HSE cell extracts information about the motion of a fly from optic flow. Previous work, which involved recording the responses of the HSE cell to simple visual stimuli (measured as a change of electrical potential of the neuron), suggested that HSE responds only to rotations of the visual world. However, the use of more natural visual stimuli suggests that some functions of the HSE cell may have been missed.[Fig pbio-0030209-g001]

**Figure pbio-0030209-g001:**
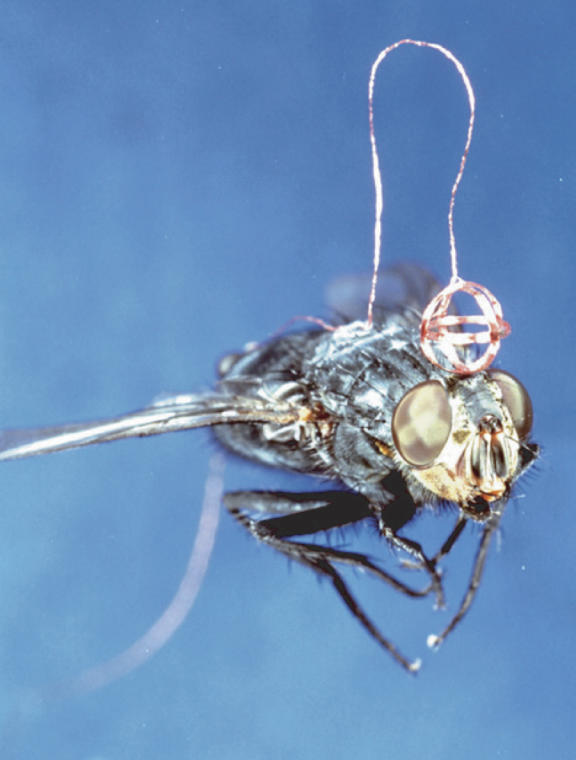
HSE neurons in the blowfly allow the fly to extract visual information about the depth structure of the world during flight

How HSE cells respond cannot be recorded in freely moving animals due to technical difficulties. To get around this problem, the Dutch group recorded the free flight of blowflies, including their characteristic head and body movements. The visual stimuli during this behavior were then reconstructed in computer-generated simulations and played back by the German group to flies in the panoramic virtual reality stimulator. Since this was done in the laboratory, the authors could record the responses of the HSE cell as the flies watched this movie.

Normal blowfly flight style involves a combination of saccadic (jerky) turns, where the head rotates at high velocity, and periods of forward motion accompanied by a constant gaze. During a playback of ten different versions of this behavior, the authors did not see a positive change in potential of the HSE cell during saccadic turns, as might be expected from previous conventional recordings. Instead, the HSE cell was depolarized by optic flow between saccades—when the fly's head was not rotating.

This surprising result suggests that blowflies may gather useful visual information about the world from translational (movement without rotation) optic flow—when their heads are not rotating and their gaze is fixed. In fact, the authors found that the blowfly's flight strategy allows information to be extracted from translational optic flow under situations where optic flow from rotation might otherwise dominate. Thus, HSE cells may not only encode information from dominant rotations of the fly itself, but allow the fly to extract “behaviorally relevant information” about the depth structure of the world. However, as the authors point out, it is not yet known whether the blowfly's nervous system can garner rotational and translation information from the combined output of HSE cells.

Nevertheless, through the use of a novel method to play back natural flight behaviors, the authors have been able to discern a new function for a well-studied motion-sensitive neuron—a function that appears to emerge from the blowfly's own behavior. Future experiments can now begin to explore how the fly uses the information generated by this new function.

